# Knock-down of the TIM/TIPIN complex promotes apoptosis in melanoma cells

**DOI:** 10.18632/oncotarget.27572

**Published:** 2020-05-19

**Authors:** Abhijit Chakraborty, Faisal Aziz, Eunmiri Roh, Le Thi My Le, Raja Dey, Tianshun Zhang, Moeez G. Rathore, Aalekhya Sharma Biswas, Ann M. Bode, Zigang Dong

**Affiliations:** ^1^The Hormel Institute, University of Minnesota, Austin, MN 55912, USA; ^2^Immunology, Allergy and Rheumatology Section, Baylor College of Medicine, Houston, TX 77030, USA; ^3^Pediatric Gastroenterology and Liver Center, Baylor College of Medicine, Houston, Texas, Houston, TX 77030, USA; ^4^College of Medicine, Zhengzhou University, Zhengzhou, Henan Province 450052, China

**Keywords:** TIMELESS, TIPIN, apoptosis, xenograft, Cryo-EM

## Abstract

The Timeless (TIM) and it's interacting partner TIPIN protein complex is well known for its role in replication checkpoints and normal DNA replication processes. Recent studies revealed the involvement of TIM and TIPIN in human malignancies; however, no evidence is available regarding the expression of the TIM/TIPIN protein complex or its potential role in melanoma. Therefore, we investigated the role of this complex in melanoma.

To assess the role of the TIM/TIPIN complex in melanoma, we analyzed TIM/TIPIN expression data from the publicly accessible TCGA online database, Western blot analysis, and RT-qPCR in a panel of melanoma cell lines. Lentivirus-mediated TIM/TIPIN knockdown in A375 melanoma cells was used to examine proliferation, colony formation, and apoptosis. A xenograft tumor formation assay was also performed.

The TIM/TIPIN complex is frequently overexpressed in melanoma cells compared to normal melanocytes. We also discovered that the overexpression of TIM and TIPIN was significantly associated with poorer prognosis of melanoma patients. Furthermore, we observed that shRNA-mediated knockdown of TIM and TIPIN reduced cell viability and proliferation due to the induction of apoptosis and increased levels of γH2AX, a marker of DNA damage. In a xenograft tumor nude mouse model, shRNA-knockdown of TIM/TIPIN significantly reduced tumor growth.

Our results suggest that the TIM/TIPIN complex plays an important role in tumorigenesis of melanoma, which might reveal novel approaches for the development of new melanoma therapies. Our studies also provide a beginning structural basis for understanding the assembly of the TIM/TIPIN complex. Further mechanistic investigations are needed to determine the complex’s potential as a biomarker of melanoma susceptibility. Targeting TIM/TIPIN might be a potential therapeutic strategy against melanoma.

## INTRODUCTION

Melanoma is the most aggressive form of skin cancer that develops when melanocytes start to grow out of control [1]. According to a study from the U. S. in 2019, an average of 96,480 new patients were diagnosed with melanoma. Among those diagnosed were 57,220 men and 39,260 women and 7,230 deaths from melanoma are expected (4,740 men and 2,490 women). Its incidence in U. S. has been increasing at a rate of about 3% per year; however, in 2019, new melanoma cases increased by 7.7% [2].

Melanoma is frequently associated with increased resistance to apoptosis induced by various therapeutic modalities. [3, 4]. As a result, conventional treatment strategies for metastatic melanoma often produce insignificant outcomes [5, 6]. Cancer chemotherapeutic efficacy is dependent on intrinsic or acquired tumor resistance to multiple drugs with different mechanisms of action [7]. Although traditional chemotherapeutic drugs theoretically target all metastatic sites, treatments do not provide the expected results. Hence, an alternative approach to drug development is urgently needed to ensure improved clinical outcomes.

TIMELESS (TIM) is a 139 kDa protein that was first characterized in Drosophila melanogaster and is required for maintenance of normal mammalian circadian rhythm, cellular metabolism, cell proliferation, DNA replication, and DNA damage repair [8–13]. TIM-interacting protein (TIPIN) is a 301-aa protein that interacts with TIM and was originally identified in yeast [14]. The exact function that TIPIN plays in mammalian cells is still unknown. Chou et al. showed that TIPIN plays an important role in cell cycle arrest in response to DNA checkpoint responses by forming a complex with TIM [15]. The TIM-TIPIN interaction also plays an important role in replication fork protection [12, 16] and participates in normal DNA replication to maintain genomic stability [11]. The TIM/TIPIN complex interacts with components of the proliferating cell nuclear antigen (PCNA) [17] and replication protein A (RPA) [18]. The TIM/TIPIN complex also has an important role in coordinating replicative helicases and polymerases to prevent the accumulation of single-stranded DNA (ssDNA) [12].

Several previous reports show the involvement of TIM in human cancers [19] of many different organs, including lung [20], breast [21–23], liver [24], prostate [25], colon [26], kidney [27], bladder [28], pancreas [29], and blood [30]. Sjoblom et al. [31] showed that TIM mutations are involved in breast cancer. In 2010, Yang et al. [32] reported that knock down of TIM induced doxorubicin-mediated cytotoxicity in colon cancer cells. They concluded that TIM is required for G2/M checkpoint control and ATM (ataxia telangiectasia mutated)-dependent CHK2 activation. Recently, a study showed that ERK activation contributes to the overexpression of TIM in colon cancer and knock down of TIM causes G2/M arrest in colon cancer [33]. In 2011, Fu et al. [21] reported that TIM may contribute to the carcinogenesis of breast cancer. Their study revealed that T single-nucleotide polymorphism (SNP) in TIM increases breast cancer risk and in 2017, Chi et al. [34] showed that TIM activated Myc, which contributes to the progression of breast cancer. A study from 2012 [20] showed that TIM overexpression is associated with poor survival of lung cancer patients. In contrast, only a very limited number of studies have been conducted to determine the role of TIPIN in cancer. Baldeyron et al. in 2015 reported that TIPIN is a potential treatment target for the worst prognosis-associated breast cancers, such as triple-negative breast cancer [35].

TIM/TIPIN has not been heavily investigated in the context of melanoma. However, the fact that all cancers share some common mechanism (e. g., all cancers have proliferation advantage compared to non-cancerous cells) coupled with the observation that this complex is associated with several different cancer types (presence of TIM mutation in breast cancer; induction of doxorubicin-mediated cytotoxicity in colon cancer cells upon TIM knockdown; activation of Myc by TIM) prompted us to speculate that TIM/TIPIN could be associated with and confer some growth advantage to melanoma cells. We conducted experiments to test this premise. No high-resolution structure of TIM/TIPIN complex is currently available. Thus, to begin to better understand the interaction between these two proteins, we determined the cryo-electron microscopic (EM) structure of human TIM-TIPIN to visualize an intermediate conformation.

The current study demonstrates that TIM and TIPIN are overexpressed in melanoma and examines the effects of short hairpin RNA (shRNA)-mediated TIM knockdown on growth, apoptosis, and tumor formation by using the A375 melanoma cell line. We found that TIM knockdown compromised proliferation, which is associated with apoptosis in melanoma cells. TIM and TIPIN depletion or just TIPIN silencing also led to an increase in H2AX phosphorylation. The findings suggest the potential of TIM as a prognostic marker and a therapeutic target for melanoma.

## RESULTS

### TIM and TIPIN are overexpressed in melanoma cells

**Figure 1:**
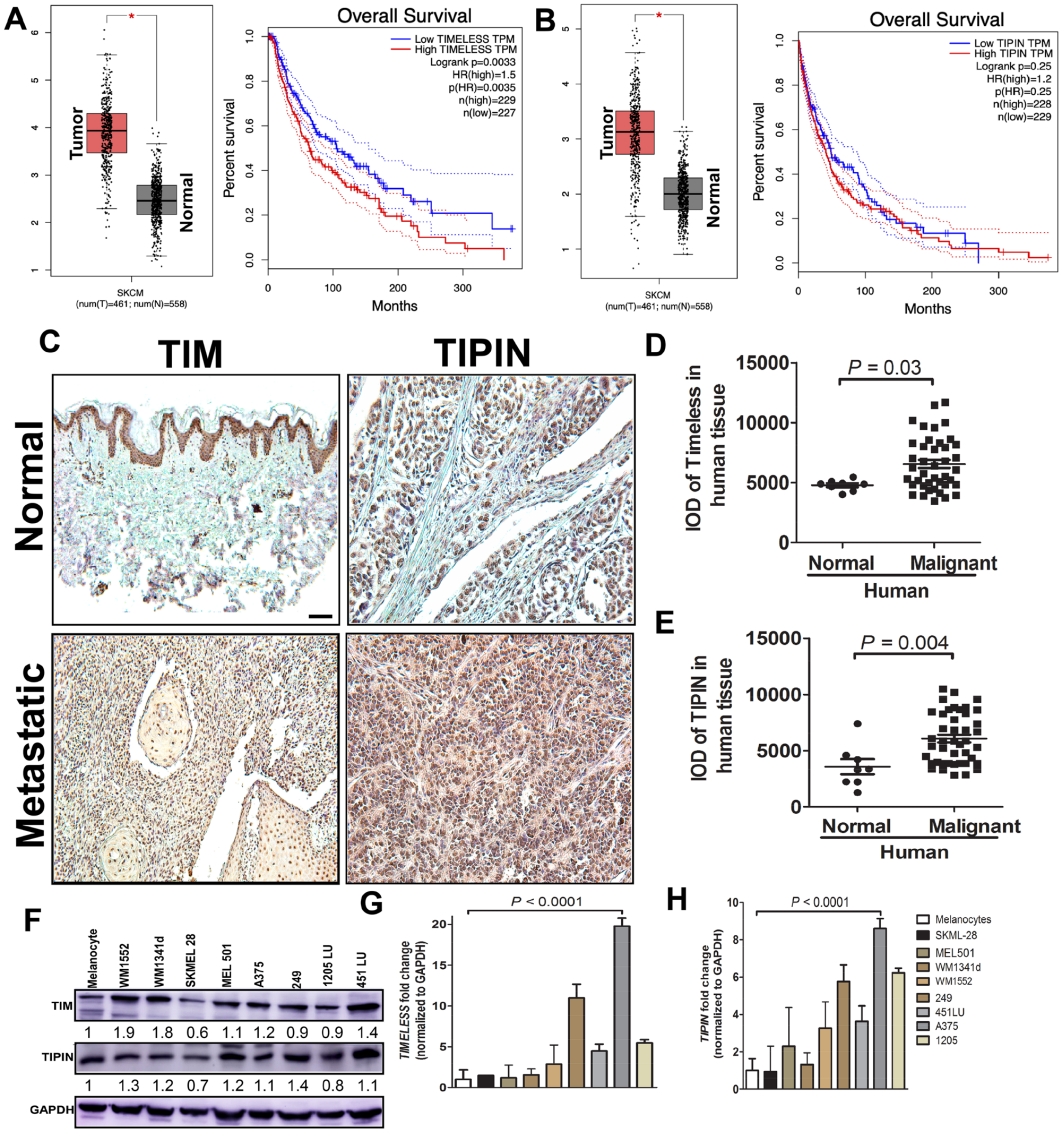
TIM and TIPIN are overexpressed in melanoma. Box plots show the average fold change in (**A**) TIM and (**B**) TIPIN and overall survival analysis from the online TCGA database http://cancergenome.nih.gov/ relative to normal (T-461, N-558) human tissue. Immunohistochemical localization of TIM (**C**, **D**) and TIPIN (C, **E**) in human melanoma metastatic (*n* = 40) and normal (*n* = 8) tissue samples. Scale bars represent 100 μm (10×). (**F**) Total protein expression of TIM and TIPIN in different melanoma cell lines. In all cases, fold changes for the protein expression was calculated using Image J software (normalized against GAPDH) and is provided beneath the panel. qPCR mRNA analysis of (**G**) TIM and (**H**) TIPIN in melanoma cell lines. Bar graphs show the average fold change in mRNA levels relative to normal cells.

To investigate the role of TIM and TIPIN in the progression of melanoma, we first analyzed TIM and TIPIN expression in 461 melanoma tissue samples and 558 normal tissue samples from The Cancer Genome Atlas (TCGA) Data Portal. GEPIA generated box plots with jitter showing differences between TIM and TIPIN expression in melanoma (TCGA tumors vs. TCGA normal). (Figure 1A, 1B). Analysis of variance was used to calculate differential expression. Overall survival (OS) was also determined based on gene expression (Figure 1A, 1B). The results show that TIM and TIPIN were significantly upregulated in melanoma tissue compared to normal tissue. High expression of TIM is associated with poor prognosis in melanoma patients, whereas TIPIN expression does not affect overall survival of patients. Tissue microarray staining also revealed that TIM and TIPIN are highly expressed in metastatic conditions compared to normal (Figure 1C–1E). Overall, these results suggest that TIM and TIPIN are upregulated in melanoma. We next evaluated TIM and TIPIN expression in a panel of human melanoma cancer cell lines (WM1552, WM1341d, SKMEL28, MEL501, A375, 249, 1205 LU, and 45 I LU). Western blot and real-time-PCR analysis showed that *TIM* and *TIPIN* were significantly elevated in melanoma cells compared with normal melanocytes (Figure 1F–1H).

### Knockdown of TIM and TIPIN suppresses melanoma cell proliferation

**Figure 2:**
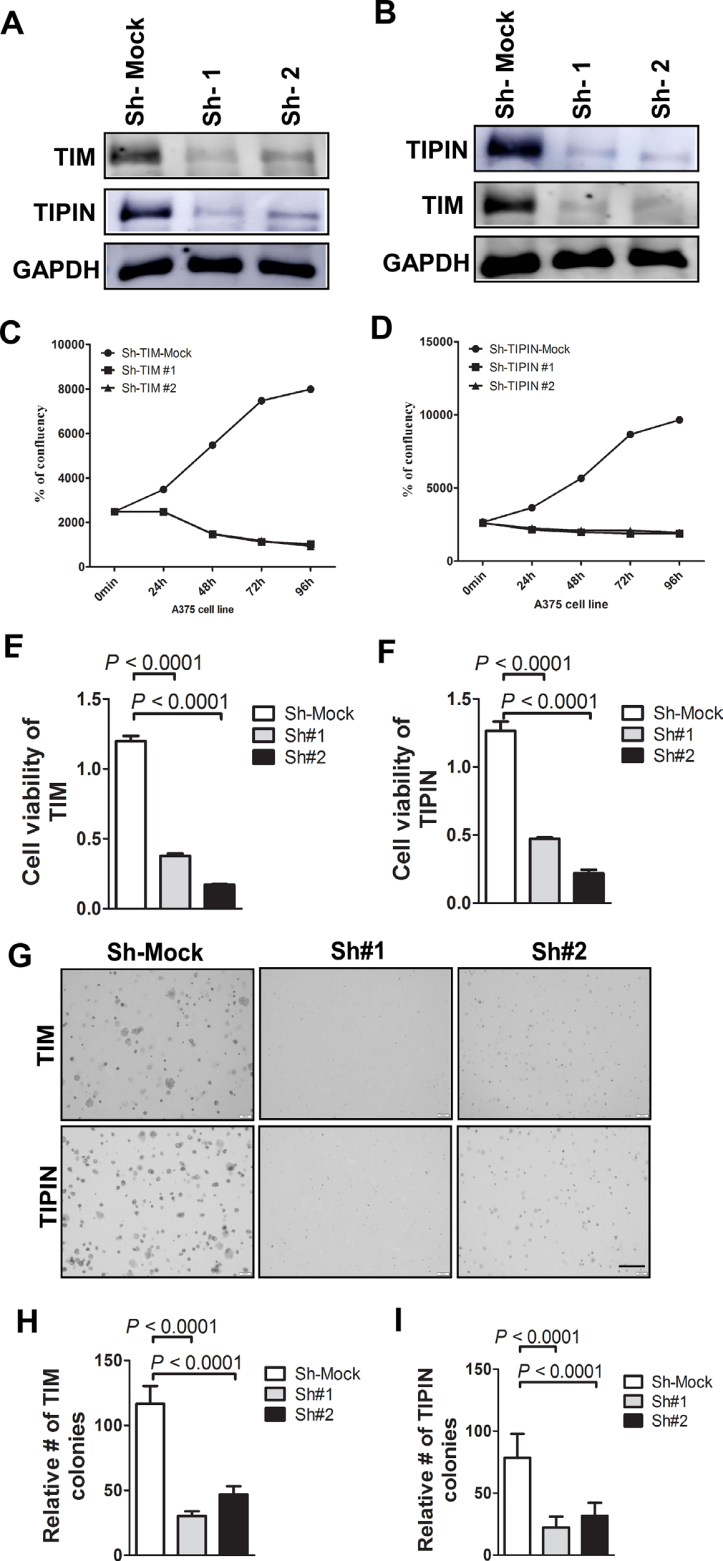
Knockdown of TIM/TIPIN inhibits melanoma cell proliferation. Western blotting was performed to analyze the knockdown effectiveness of (**A**) TIM and (**B**) TIPIN in melanoma cells. Proliferation of A375 melanoma cells was determined using IncuCyte S3 imaging (Essen BioScience, serial number = IC50471) after transfection with shRNA against (**C**) TIM or (**D**) TIPIN relative to mock control. Viability of A375 cells was determined after knockdown of (**E**) TIPIN or (**F**) TIM. (**G**) Representative images photographed at 10× magnification for soft agar colony formation assay are shown. (**H**) TIM and (**I**) TIPIN represent means ± standard deviations from triplicate determination for soft agar colony formation.

To evaluate the role of TIM and TIPIN expression in the pathogenesis of melanoma cells, a transient knockdown of TIM and TIPIN by shRNA in the A375 cell line was performed (Figure 2A, 2B). To minimize the possibility of off-target effects, we used two pairs of non-overlapping synthesized oligos targeting TIM or TIPIN. Then we examined the effect of TIM knockdown on cellular proliferation using the IncuCyte^®^ Cell Count Proliferation Assay (Figure 2C, 2D) as well as a crystal violet assay for determining viability (Figure 2E, 2F). Next, to evaluate the effects of knockdown of these two genes on anchorage-independent growth, we conducted a soft agar colony formation assay (Figure 2G–2I). All findings suggest that TIM and TIPIN knockdown decreases cell proliferation and suppresses growth.

### TIM depletion induces apoptosis in melanoma cells through DNA damage

**Figure 3:**
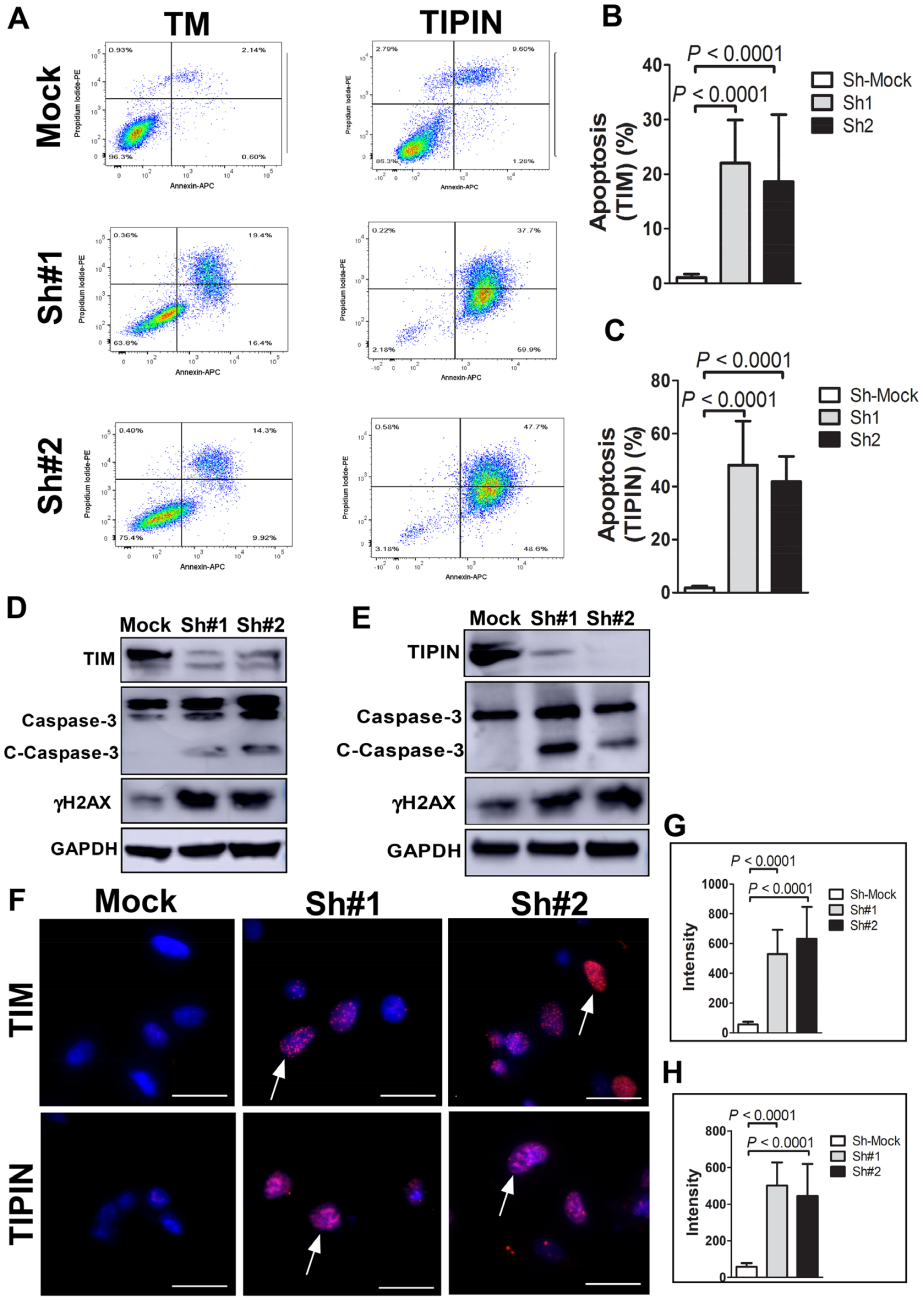
Knockdown of TIM or TIPIN induces apoptosis of melanoma cells. (**A**) Cells transfected with TIM- (left panels) or TIPIN- (right panels) shRNA were double-stained with annexin V-FITC and PI and then subjected to flow cytometric analysis. Quantitation of percentage of apoptotic cells expressing knock down of (**B**) TIM or (**C**) TIPIN (PI negative and Annexin V positive staining). The values are expressed as means ± S. E. M. of 3 separate experiments. Melanoma cells were transfected with (**D**) TIM-shRNA or (**E**) TIPIN-shRNA and cell lysates were prepared after 48 h. The expression levels of TIM, TIPIN, total and cleaved caspase-3, and γH2AX were detected by Western blotting with GAPDH as the internal control. (**F**) A375 cells were transfected with the indicated shRNAs and immunofluorescence was conducted. Cells were incubated with an γH2AX antibody and the nuclei were stained with DAPI. The percentage of cells showing at least five γH2AX foci (indicated by arrows) were counted and greater H2AX phosphorylation was observed in TIM- or TIPIN-knockdown cells. Quantitative analysis was conducted to measure the intensity of (**G**) shTIM and (**H**) shTIPIN-transfected cells relative to mock-control.

We performed additional experiments to determine whether TIM knockdown leads to apoptosis in melanoma cells. We assessed apoptosis induction at 96 h post-transfection in A375 cells. Staining with annexin V confirmed that TIM and TIPIN depletion induced apoptosis in both cell lines (Figure 3A–3C). Similar results were obtained when we analyzed the levels of cleaved-caspase 3 by immunoblotting (Figure 3D, 3E). Altogether, our data suggest that TIM and TIPIN knockdown leads to apoptosis in melanoma cells. Western blot and immunofluorescence results revealed that TIM and TIPIN knockdown was associated with an increase in phosphorylated histone H2AX (γH2AX; Figure 3D–3H), which is consistent with previous findings [15].

### Knockdown of TIM/TIPIN inhibits tumor growth of melanoma cells in a nude mouse xenograft model *in vivo*

**Figure 4:**
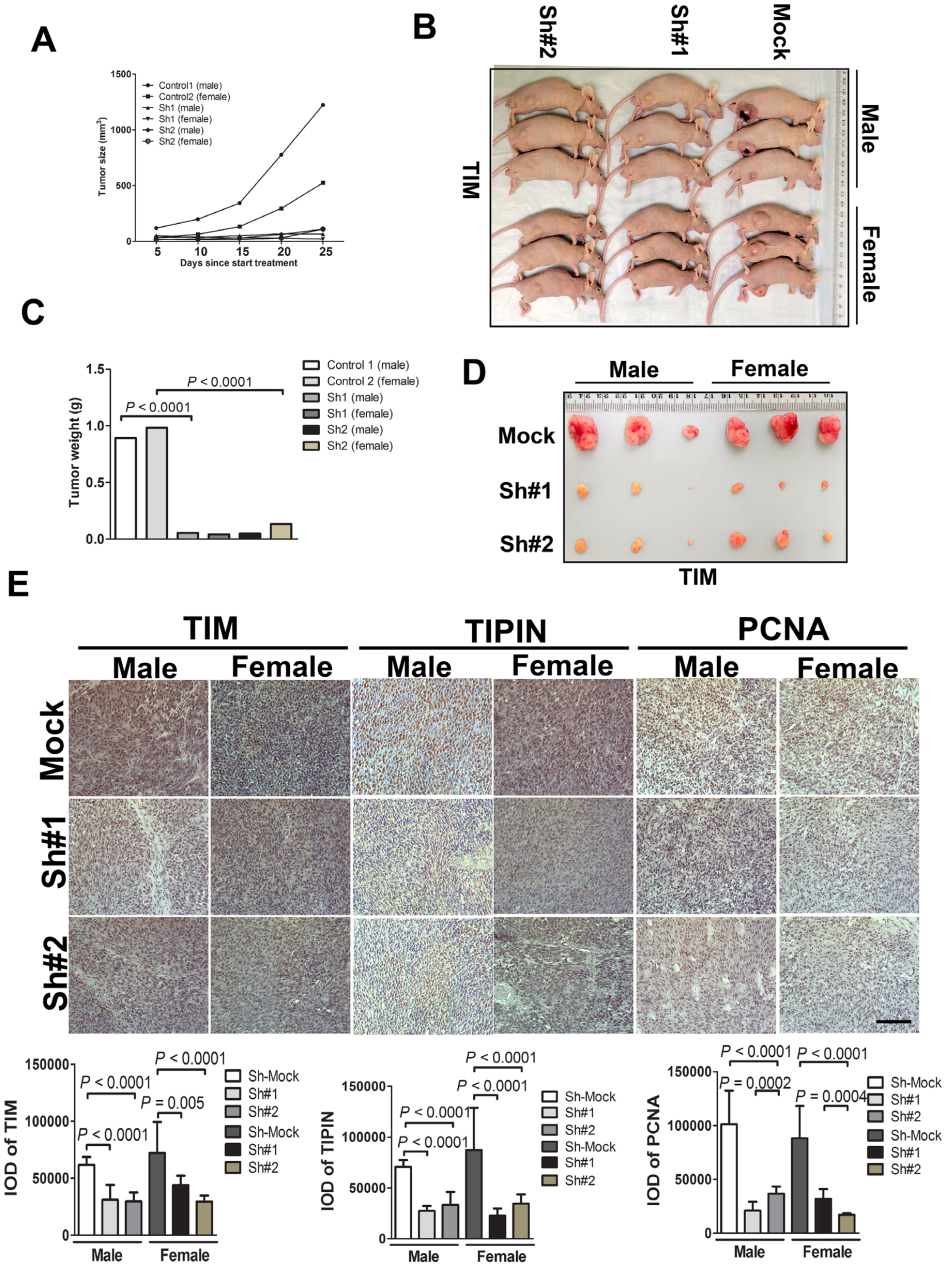
TIM knockdown diminishes tumorigenicity of melanoma cells *in vivo*. Melanoma cells transfected with the non-silencing shRNA (mock sequence) as a negative control or transfected with TIM shRNA were inoculated subcutaneously into male and female mice. (**A**) Graph showing tumor size measured every 5 days. (**B**) Representative photographic images of nude mice showing the growth of the xenograft tumors. (**C**) Graph showing the mean tumor weight of the resected tumor. (**D**) Images of xenograft tumors dissected from the nude mice 4 weeks after subcutaneous inoculation. (**E**) Tumor tissues were subjected to immunostaining for determination of TIM, TIPIN, and PCNA expression. Quantitation of histochemical analysis of TIM, TIPIN, and PCNA in tissue samples from tumor xenografts mice.

**Figure 5:**
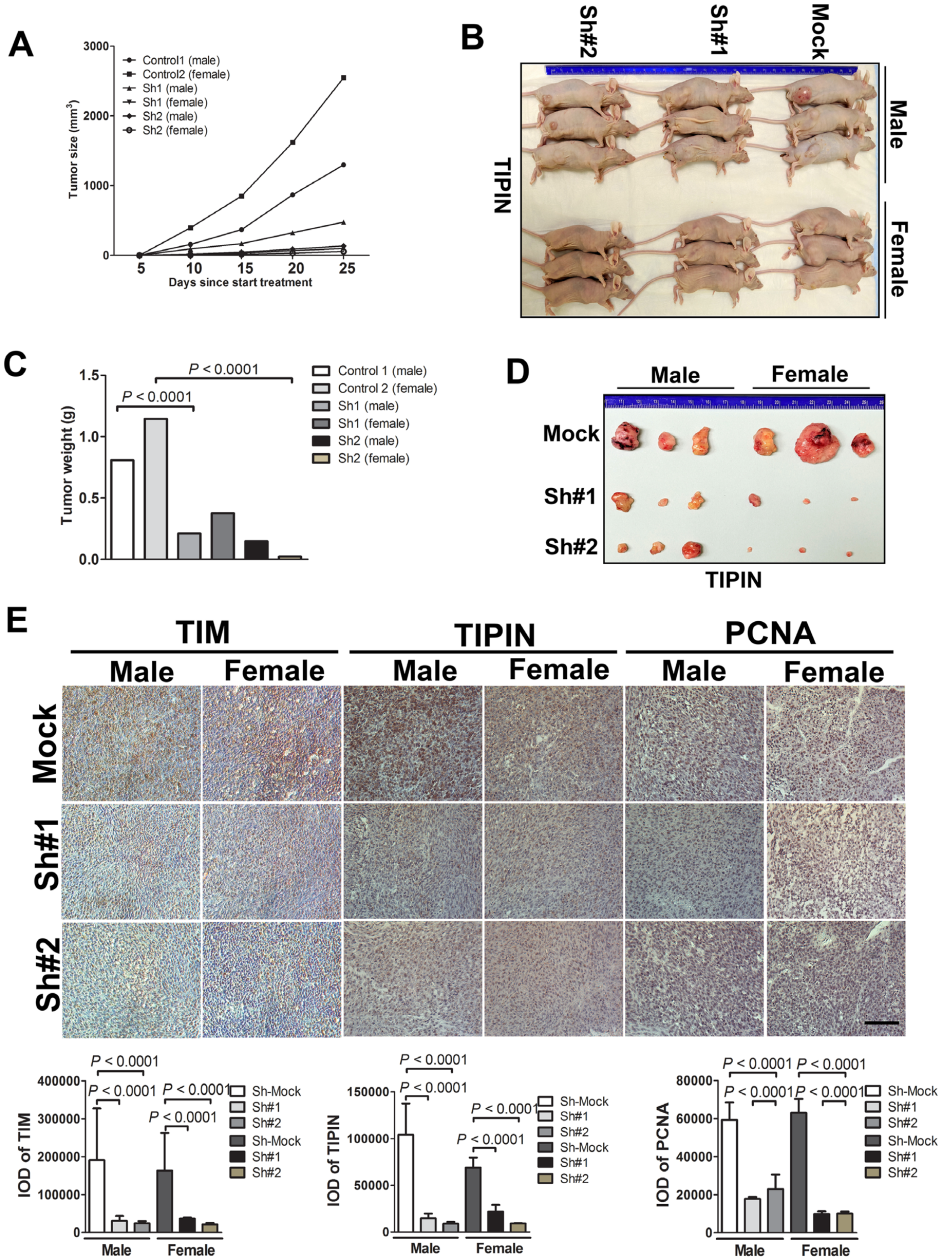
TIPIN knockdown diminishes tumorigenicity of melanoma cells *in vivo*. Melanoma cells transfected with a non-silencing shRNA (mock sequence) as a negative control or transfected with TIPIN shRNA were inoculated subcutaneously into male and female mice. (**A**) Graph showing tumor size measured every 5 days. (**B**) Representative photographic images showing the growth of the tumors in nude mice and (**C**) graph showing the mean tumor weight of the resected tumor. (**D**) Images of xenograft tumors dissected from nude mice at 4 weeks after subcutaneous inoculation. (**E**) Tumor tissues were subjected to immunostaining for determination of expression of TIM, TIPIN, and PCNA expression in the tumor xenografts. Quantitation of histochemical analysis of TIM, TIPIN, and PCNA in tissue samples from tumor xenografts mice.

To determine the effect of TIM and TIPIN on the tumorigenic capacity of melanoma cells, we investigated the effects of TIM and TIPIN loss on tumor growth *in vivo* by using shTIM- and shTIPIN-transfected A375 cells. TIM and TIPIN depletion diminished the tumorigenicity of melanoma xenografts in immunocompromised mice (Figures 4, 5). Tumors formed by shMock cells were much larger than those tumors generated by the TIM-knockdown cells as well as the TIPIN-knockdown cells. Overall, these results suggest that TIM and TIPIN promote tumorigenicity of melanoma cells *in vivo*.

We further explored the potential mechanism of TIM/TIPIN knockdown on proliferation. Immunohistochemistry was performed on tumor tissues, and interestingly, the results showed that the expression level of PCNA in the TIM- and TIPIN-knock down groups was significantly reduced, suggesting a marked inhibition of cell proliferation (Figure 4E and Figure 5E). These data demonstrated that TIM and TIPIN are involved in the proliferation of melanoma cells *in vivo*.

### Electron microscopic reconstruction of the TIM/TIPIN complex

**Figure 6:**
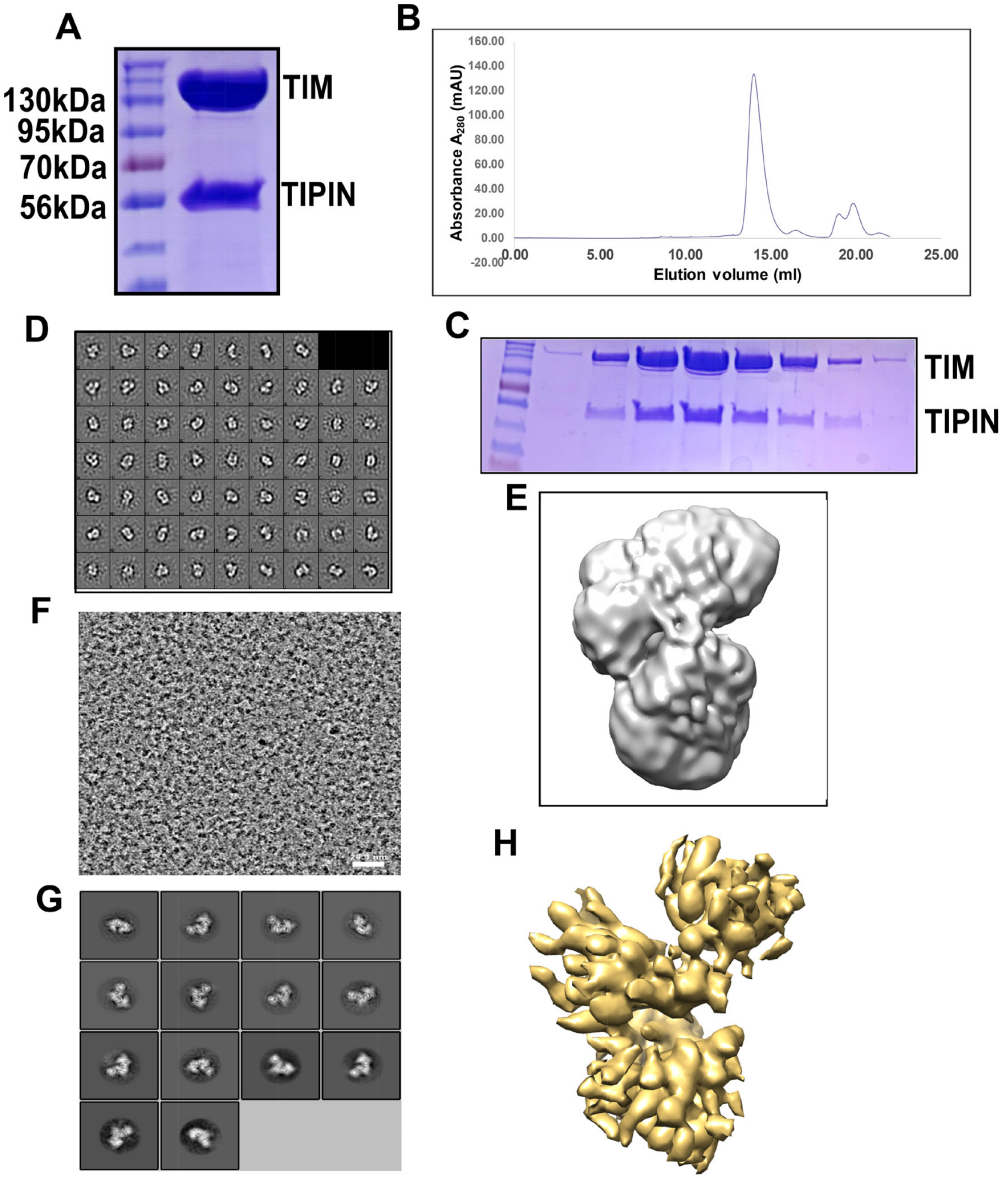
Reconstitution of the TIM/TIPIN complex visualized by cryo-EM. (**A**) An SDS-PAGE gel of the purified TIM/TIPIN complex with the protein indicated on the right. (**B**) Representative gel-filtration chromatography of the TIM-TIPIN complex. The fractions of proteins were collected manually and the absorption was monitored at 280 nm wavelength. (**C**) The peak fractions were visualized by SDS-PAGE and Coomassie blue staining. The apparent protein sizes correspond well to the expected sizes for TIM (170 kDa including the fused 3xFLAG-tag) and TIPIN (56 kDa). The TIM/TIPIN complex is observed in the peak fraction corresponding to an elution volume of 14.23 ml. Proteins are indicated to the right. Note that only gel filtration peak fractions are shown. (**D**) 2D class averages show that the TIM/TIPIN complex adopts elongated particles with a well-defined shape. (**E**) A 3D map of the TIM/TIPIN complex was created from 2D-classes of negative stain by using cisTEM. (**F**) A raw EM image of the TIM/TIPIN complex. The scale bar corresponds to 28.4 nm. (**G**) Representative 2D class averages from cryo-EM that are consistent with the particle shapes derived from negative staining. (**H**) A good 3D map (7.3 Å) of the TIM/TIPIN complex was created from the 2D-class averages from cryo-EM by using cisTEM.

The TIM/TIPIN complex was reconstituted by co-expression in mammalian (293F) cells and then purified. We observed that the full length TIM or TIPIN was only stable when they were co-expressed and co-purified, which supports results of previous studies [36, 37]. We purified the TIPIN and TIPIN complex using FLAG-M2 beads (Figure 6A). Size exclusion chromatography of the TIM/TIPIN complex gives a single peak, showing a stoichiometry of 1:1 (Figure 6B). The peak fractions were visualized by SDS-PAGE and Coomassie blue staining (Figure 6C). The apparent protein sizes correspond well to the expected sizes for TIM (170 kDa including the fused 3xFLAG-tag) and TIPIN (56 kDa). The TIM/TIPIN complex is observed in the peak fraction corresponding to an elution volume of 14.23 ml. Proteins are indicated to the right. The presence of TIM and TIPIN in the complex was determined by Western blot analysis (Supplementary Figure 1A, 1B). Complex stability was further confirmed by native page gel electrophoresis and Coomassie blue staining (Supplementary Figure 1C). To obtain insights into the architecture of the TIM/TIPIN complex, we observed the complex by negative stain EM (Figure 6D). The electron micrographs showed a stable, monodispersed complex formation of the TIM/TIPIN complex without any aggregation. Initially, we imported 92 images collected from the negative stained grid within the cisTEM GUI to create a map of the TIM/TIPIN complex (Figure 6E). Amazingly the shape of one of the predicted models of TIM which was generated using I-tasser online server fairly agrees with the shape of the final map generated from the negative stained EM-data (Supplementary Figure 1D).

Following the analysis of the negative stained EM-data, we processed cryo-EM data collected by our in-house Titan Krios electron microscope with the Falcon III detector. using MotionCor2 [38] within Relion 3.0.6. The 2001 integrated images were then imported to cisTEM for contrast transfer function (CTF) estimation using Find CTF, particle picking by Find Particles, 2D classification by 2D Classify, initial model building using Ab-Initio 3D, and finally the refinement of the map generated was performed by Auto Refine module within the cisTEM GUI. Following CTF estimation using Find CTF [38–40], we selected 1553 good images through the inspection of the thon ring, amplitude spectrum and quality of fit, and the micrograph itself on the same graphical window for each integrated image. A screenshot for one of the selected images is depicted in Supplementary Figure 1E showing the thon ring, amplitude spectrum and quality of fit, and the micrograph itself. A total of 335,788 particles were picked using Find Particles [41]. A screen shot of the image is shown in Figure 6F and a particle picking image is shown in Supplementary Figure 1F. Initial 2D classification was performed using an unbinned dataset with a pixel size of 0.709Å. The 2D classes with averages showing no clear secondary structure were discarded. The remaining 49,502 particles after selecting best class averages (Figure 6G) were subjected to Ab-Initio 3D classification into 3 classes followed by refinement using the Auto Refine module in cisTEM. Finally, a 7.3 Å map (Figure 6H) was generated showing some extra density for the TIPIN molecule. A similar shaped map (Supplementary Figure 1G) has also been generated using Relion 3.0.6 thereby relying on the map generated by the cisTEM software. Because of the low resolution, we did not attempt to build an atomic model on this map.

## DISCUSSION

Uncontrolled growth and invasiveness are two major hallmarks of malignancy [42]. In this study, we characterized the role of TIM and TIPIN in melanoma. To the best of our knowledge, this is the first report focusing on the role and function of this two-protein complex in human cancer. We found that the levels of these two protein in cells are interdependent because depletion of one protein led to the depletion of the other. This suggests that depletion of either protein produces a similar phenotype. This is a primary reason behind the difficulty in separate examination of the specific functions of the individual proteins.

We conducted a series of studies to examine the role of the TIM/TIPIN complex in melanoma. The boundless growth of tumors is considered to be a result of infinite proliferation, defective apoptosis, and deregulation of cell cycle. Our series of *in vitro* and *in vivo* tumorigenesis studies revealed that the proliferation potential of melanoma cells was significantly inhibited after knockdown of *TIM* and *TIPIN*.

The high expression of TIM and TIPIN in multiple cancers suggests that this complex may promote cell proliferation by inducing DNA synthesis, DNA damage repair, and cell cycle advancement by numerous mechanisms as described in previous publications [11, 12, 16, 34, 37, 43–46]. Our study demonstrated that the TIM/TIPIN complex is abundantly expressed in melanoma compared with normal melanocytes. Our survival analysis revealed that high TIM/TIPIN expression is an independent biomarker associated with poorer clinical outcomes in melanoma patients. Our findings supported other work where scientists reported the importance of TIM/TIPIN in many different types of human cancers [17, 20–23, 25–30, 32]. TIM expression in breast cancer was also reported and TIM was found to be is a promising marker of tamoxifen-resistant estrogen receptor α-positive breast tumors [21–23]. In the case of TIPIN in breast cancer, TIPIN knockdown in triple negative breast cancer cells decreased tumorigenicity *in vitro* and delayed tumor growth *in vivo* [35]. In cases of hepatocellular carcinoma (HCC), overexpression of TIM exerted oncogenic function through CHEK2 and eukaryotic elongation factor 1A2 (EEF1A2) [24].

TIM expression is also associated with clinicopathological factors in bladder cancer. This study reveals that TIM is upregulated in cancer tissues compared to normal urothelial biopsies and *TIPIN* mRNA levels exhibited a similar trend although the increased protein expression in cancer tissue was more modest [28]. On the other hand, TIM is downregulated in kidney cancer cells [27] and pancreatic ductal adenocarcinoma [29]. Mechanistically, TIM regulates tumor proliferation, apoptosis, metastasis, and cell cycle and also has been implicated in proliferation of leukemia stem cells [30]. Consistent with the findings from other studies [15, 47], we also found that knock down of TIM and TIPIN compromised proliferation of melanoma cells. Because the TIM/TIPIN complex is involved in DNA replication fork stability, we investigated the mechanisms behind the melanoma cells to undergo apoptosis following TIM or TIPIN deletion. We found that TIM or TIPIN deletion led to increased H2AX phosphorylation, which supported data shown in previous studies [14, 33, 44]. We found that the proliferation-related antigen PCNA was substantially reduced after TIM/TIPIN knockdown *in vivo*. According to previous studies, PCNA is a protein that is present in the cell proliferative phase and one of the markers of proliferating cells [48]. Similarly, our study also showed that TIM/TIPIN knockdown could inhibit cell colony formation.

Moreover, we found that TIM and TIPIN expression is highly associated with melanoma. Thus, the TIM/TIPIN complex might be an effective biomarker for melanoma and for determining optimal treatments in melanoma patients. Our present study supports some of the previous studies where TIM was reported to play an oncogenic role in HCC and promote tumor cell migration [24] and was also associated with prostate tumor metastasis [25]. Our results also are in agreement with the study of Zhang et al., wherein they reported that TIM expression is clearly associated with survival of cervical cancer patients [49]. Furthermore, we found a strong association between poorer survival and high TIM/TIPIN protein expression in patient tissues and also the importance of the TIM/TIPIN complex in cell proliferation. Thus, TIM and TIPIN might be a potential marker for melanoma and a good prognostic marker for melanoma patients, although further studies are required.

The information regarding the role of the TIM/TIPIN complex in cancer is very limited. The possible mechanisms are also unknown as well, and thus, the current manuscript has shed some light in this area. Additional cell lines should be used for further investigations. Also, the identification of additional genes and proteins that could be upregulated or downregulated should be examined further. Inhibitors or activating agents could be used to further study the effects of the TIME/TIPIN complex on associated signaling pathways.

TIM and TIPIN form a stable complex and serve as a key player in controlling melanoma. In this study, we purified the human TIM-TIPIN complex to homogeneity and determined the cryo-EM structure. The study provides a structural basis for understanding the assembly of the complex. A high resolution Cryo-EM map of this protein complex is being examined and could be helpful to design an inhibitor in the near future. Overall, the work presented herein provides new avenues for the development of more potent antibodies and small-molecule inhibitors of the TIM/TIPIN complex that could be used alone or in combination with small-molecule inhibitors, which could be a promising drug to treat melanoma in the future.

In summary, our study demonstrates that *TIM* and *TIPIN* each are overexpressed in melanoma cells, and could regulate proliferation and migration of melanoma cells. This study also suggested an important role of this complex in the progression of melanoma. These results represent a new area for molecular research focusing on melanoma, and also provide experimental data for further study. These data indicate that TIM and TIPIN represent promising targets for anticancer therapies. Moreover, they represent potentially valuable prognostic markers for melanoma patients. Further investigations of cryo-EM TIM/TIPIN structural analysis with a high-resolution model are necessary for designing future promising drugs in melanoma treatment.

## MATERIALS AND METHODS

### Plasmids, shRNA

pcDNA4-Flag-Timeless (Addgene plasmid # 22887; http://n2t.net/addgene:22887; RRID: Addgene_22887) and pcDNA3-Flag-Tipin Sancar (Addgene plasmid # 22889; http://n2t.net/addgene:22889; RRID: Addgene_22889) were gifts from Aziz Sancar [37, 50]. The short hairpin RNA to knock down the *TIM* and *TIPIN* genes was obtained from the University of Minnesota genomics center (http://genomics.umn.edu/) and the sequence for shTIM and shTIPIN is shown in Supplementary Table 1.

### Reagents and antibodies

The antibodies to detect TIM (A300-960A) were obtained from Bethyl Laboratories (TX, USA). The antibodies to detect β-actin (sc-47778), GAPDH (sc-3223), or TIPIN (sc-135580) were purchased from Santa Cruz Biotechnology, Inc. (Santa Cruz, CA, USA). Antibodies to evaluate p-γH2AX (2577S), PCNA (13110), or cleaved caspase-3 (9661) were purchased from Cell Signaling Technology (Danvers, MA, USA). The TIPIN (anti-FLJ20516) antibody for IHC was from Abnova (Taiwan) and the apoptosis Western blot cocktail (ab136812) was purchased from Abcam (UK).

### Cell culture and transfection

The melanoma cell lines (melanocytes, WM 1552, WM 1341d, SKMEL28, MEL501, A375, 249, 1205LU, 451LU), and 293T cells were purchased from American Type Culture Collection (ATCC; Manassas, VA, USA). For protein purification, 293-F cells (FreeStyle, R79007) were used and purchased from Thermo Fisher Scientific, Waltham, MA, USA. Following ATCC protocols, all cells were cultured in a 5% CO_2_ humidified incubator at 37°C. MEL501, A375, 249, 1205LU, WM1552, 451LU, and 293T cells were cultured with DMEM containing 10% fetal bovine serum (FBS) and 1% antibiotics. SKML28 cells were cultured in minimal Eagle’s medium (MEM) supplemented with 10% FBS and 1% antibiotics and 293T cells were cultured at 37°C in a humidified incubator with 5% CO_2_ in Dulbecco’s modified Eagle’s medium supplemented with 10% FBS and 1% antibiotics. WM1341D cells were maintained in RPMI 1640 supplemented with 10% FBS. The 293F cells were cultured in FreeStyle™ 293 Expression Medium.

### Lentiviral transductions

To knock down TIM and TIPIN in A375 cells, the lentiviral vectors against TIM (sh1 & sh2), shTIPIN (sh1, sh2), pLKO.1-puro shRNA (shMock), control plasmid DNA, and packaging vectors (pMD2.0G and psPAX, Addgene Inc., Cambridge, MA, USA) were transduced into 293T cells by using iMFectin PolyDNA Transfection Reagent (GenDepot, Barker, TX, USA) following the manufacturer’s suggested protocols. The transfection reagents were incubated with 293T cells in complete growth medium overnight, and then fresh medium with antibiotics (penicillin/streptomycin) was added. Viral supernatant fractions were collected at 48 h after transfection and filtered through a 0.45 μm syringe filter followed by infection into the appropriate cells together with 10 μg/mL polybrene (Sigma-Aldrich, St. Louis, MO, USA). At 16 h after infection, the medium was replaced with fresh complete growth medium containing the appropriate concentration of puromycin (2 μg/mL). At 3 to 4 days after infection, the selected cells were used for experiments.

### Crystal violet staining assay

Cells (2 × 10^4^ per well) for measuring proliferation were seeded into 24-well plates. After overnight incubation, cells were treated with shRNA (Mock, sh1, sh2). Cells for measuring proliferation were incubated for 24, 48, or 72 h. After washing 3 times with phosphate buffered saline (PBS), cells from each different time point were fixed with methanol for 10 min and stained with 0.2% (w/v) crystal violet in 2% (v/v) ethanol for 10 min. Then, 0.5% (w/v) sodium dodecyl sulfate in 50% (v/v) ethanol was added to each well. Absorbance was measured at an optical wavelength of 540 nm using the Thermo Multiskan plate-reader (Thermo Fisher Scientific, Waltham, MA, USA).

### Cell proliferation and viability assay

Cells (1 × 10^3^ cells/well) were seeded in 96-well plates. After 24 h, cells were treated with shTIM and shTIPIN and incubated at 37°C for 24, 48, 72, or 96 h. The IncuCyteS3 live-cell imaging system (Essen BioScience, Tokyo, Japan, IC5047) was used to monitor cell proliferation and the IncuCyteS3:2019 software was used to quantify proliferation. The cell proliferation assay was performed in 6 replicates.

### Soft agar colony formation assay

Sh-TIM, sh-TIPIN knockdown cells or control cells were suspended in medium containing 0.4% agar and overlaid on 1% agar in each well of a six-well plate (2,000 cells/well), respectively. After 14–21 days, colonies were counted and photographed. The results are expressed as means ± S. D. of triplicate counts within the same experiment.

### Quantitative real-time PCR

The commercial RNA extraction kit (Ambion, Life Technologies, Van Allen Way Carlsbad, CA, USA) was used and cDNA synthesized using the amfiRivert cDNA synthesis platinum master mix (GenDEPOT, Katy, TX, USA, Cat number R5600-200). Real-time PCR (qRT-PCR) was conducted using a 7500 FastDX instrument (Applied Biosystems, MA, USA) and the power SYBR green PCR master mix (Applied Biosystems, Warrington, WA1 4SR, UK, Cat number 4367659). Primer IDs and sequences are shown in Supplementary Table 2.

### Immunofluorescence (IF) staining analysis

For cell staining, A375 cells expressing shTIM (shMock, sh1, sh2) and shTIPIN (shMock, sh1, sh2) were incubated with the primary antibody. One day later, Alexa Fluor 488-labeled or Alexa Fluor 568-labeled secondary antibodies (Thermo Scientific) were added. The DAPI-Fluoromount-G^®^ solution (Electron Microscopy Sciences, Hatfield, PA, USA) was added for nuclei staining of the cells. Fluorescence-labeled proteins were analyzed using the Zeiss fluorescent microscope with the apotome attachment and Zeiss software and Axiocam cameras (Carl Zeiss Microscopy GmbH, Deutschland).

### Apoptosis analysis by flow cytometry

For apoptosis assessment, an Annexin V-FITC (BD Pharmingen BD Biosciences, Franklin Lake, NJ, USA, Cat number 556419) binding assay was performed in A375 cells expressing shTIM (shMock, sh1, sh2) and shTIPIN (shMock, sh1, sh2). Data were analyzed with BD FlowJo version 10 software.

### Immunohistochemistry (IHC)

Commercially available patient tissue arrays from US Biomax Inc. (Rockville, MD), were used for IHC. For mouse studies, paraffin-embedded melanoma tissues were analyzed by immunohistochemistry (IHC). Serial sections (4–6 mm each) were deparaffinized in xylene and rehydrated in an alcohol concentration gradient and evaluated with antibodies to detect TIM (1:200), TIPIN (1:30 each), and PCNA (1:200). Sections were subsequently incubated with their respective secondary antibodies for 30 min at room temperature. The signal was visualized with peroxidase-labeled streptavidin complexes and DAB, and the sections were briefly counterstained with hematoxylin. The immunohistochemical localization pattern was also recorded by digital imaging (Nikon Ti-DS, Japan). The IOD value of each tumor tissue was analyzed from 3 different fields using the Image-Pro PLUS (v.6) computer software program following an established protocol.

### Western blot analysis

To determine protein concentration, a protein assay kit (Bio-Rad Laboratories, Hercules, CA) was used. After subjection to SDS-PAGE, the samples were transferred to polyvinylidene difluoride (PVDF) membranes (EMD Millipore Corporation). Then the membranes were blocked with 5% nonfat milk for 1 h at room temperature and incubated with specific primary antibodies overnight at 4°C. Finally, the membranes were incubated with a horseradish peroxidase (HRP)-conjugated secondary antibody for 1 h at room temperature. Protein bands were visualized with a chemiluminescence reagent (GE Healthcare Biosciences).

### Mouse xenograft model

Athymic nude mice (6–7 weeks) were obtained from Charles River and maintained under specific pathogen-free conditions. Mice were divided into 3 groups (*n* = 6 mice in each group) and shMock, Sh1 and sh2. A375 melanoma cells (2 × 10^6^/0.1 mL) were injected subcutaneously into the right flank of each mouse. Body weights and tumor measurements were performed once a week and tumor volume were calculated based on the formula: length × width × width × 0.52. At the end of the experiment, mice were euthanized, and tumors were harvested and fixed in formalin for further analysis. All animal studies were performed following the guidelines approved by the University of Minnesota Institutional Animal Care and Use Committee (Protocol ID: 1803-35739A). The tumor tissues were fixed and embedded in paraffin for histological analysis and immunohistochemistry staining. The IOD value was analyzed from 3 different fields of each tumor tissue by using the Image-Pro PLUS (v.6) computer software program following an established protocol.

### Protein purification

TIM and TIPIN plasmids were co-transfected into 293F cells. After 72 h, the cell pellet was rinsed in PBS and disrupted in lysis buffer (50 mM HEPES pH 7.8, 300 mM NaCl, 1 mM EDTA, 1 mM DTT, 0.5% (v/v) NP-40, 10% (v/v) glycerol, and protease inhibitor cocktail (Sigma-Aldrich), on ice for 30 min and then sonicated (Sonic, Dismembrator, Fisher Scientific, NH, USA). Whole cell lysates were centrifuged at 4°C for 30 min at 30,000 rpm using a high speed centrifuge (Sorvall Lynx6000 centrifuge, Thermo Scientific, Waltham, MA, USA) and the supernatant fractions were incubated at 4°C on a rotator mixer overnight with ANTI-FLAG^®^M2 Affinity Gel (Sigma-Aldrich). After incubation, the affinity gel was washed 3 times with (10 mL) wash buffer 1 (50 mM HEPES, pH 7.8, 500 mM NaCl, 1 mM EDTA, 1 mM DTT, 0.02% (v/v) NP-40, 10% (v/v) glycerol, and protease inhibitor cocktail; Sigma-Aldrich) and wash buffer 2 (50 mM HEPES pH 7.8, 300 mM NaCl, 1 mM EDTA, 1 mM DTT, 0.02% (v/v) NP-40, 10% (v/v) glycerol, and protease inhibitor cocktail; Sigma-Aldrich). An incubation step was performed with 5 mM ATP and 10 mM MgCl_2_ diluted in wash buffer 2 to remove chaperone protein binding contamination. Afterwards, the affinity gel was washed 3 times in wash buffer 2. The affinity-bound flag-TIM and flag-TIPIN protein complex was eluted by adding 5 volumes of elution buffer containing 100 μg/ml of 3× FLAG^®^ peptide (Sigma-Aldrich), 50 mM HEPES pH 7.8, 150 mM NaCl, and 0.5 mM TCEP (TCEP25, 51805-45-9, GOLDBIO, St. Louls, MO) and rotated at 4°C for 1 h each cycle, repeating 3 times. For every cycle, samples were centrifuged at 700 × g for 3 min and supernatant fractions containing the eluted proteins were collected. The eluted proteins were separated by SDS–polyacrylamide gel electrophoresis and stained with Coomassie blue solution (Coomassie R-250, 50% methanol, and 10% acetic acid).

### Size exclusion chromatography (SEC)

The TIM/TIPIN protein complex was further purified using size exclusion chromatography to verify the stability of the complex. The samples were purified on a Superose 6 size exclusion column (GE Healthcare, Marlborough, MA, USA) in 50 mM HEPES, pH 7.8, 150 mM NaCl, and 0.5 mM TCEP. The fractions corresponding to the TIM/TIPIN complex were eluted, collected into a new Eppendorf tube, and loaded onto an SDS–polyacrylamide gel for further SDS–PAGE and stained with Coomassie blue stain solution and the presence of both proteins was confirmed by immunoblot (IB) analysis using TIM and TIPIN antibodies.

### Transmission electron microscopy imaging: negative staining and cryo-EM data collection.

We have used the I-tasser online server (https://zhanglab.ccmb.med.umich.edu/I-TASSER/) to generate a predicted model of TIM and TIPIN separately to get an idea about the shape of the individual molecules. Our in-house Leica ACE 600 was used to perform thermal evaporation of carbon to create thin layer supports for both negative stained and cryo-EM grids. The protein complex of TIM and TIPIN was chemically fixed with 0.075% glutaraldehyde and incubated at 4°C overnight. Three microliters of sample at a concentration of 60 μg/mL was placed on a 400 mesh copper grid coated with a Formvar/carbon film (EMS400-Cu, Electron Microscopy Sciences) for 1 min. The grid was washed with 2 to 3 drops of 5 μL distilled water. Excess water was removed with Whatman filter paper. The sample was negatively stained with 3 μL 0.5% uranyl acetate (SPI Supplies, PA, USA) for 30 s. Excess staining solution was removed with Whatman filter paper, before the grid was slowly dried at room temperature. A total of 92 electron micrographs were collected using the Technai G2 Spirit BioTwin electron microscope (FEI) with a Gatan Ultrascan camera (Gatan, Pleasanton, STATE) at an accelerating voltage of 120 kV and a nominal magnification of 68,000 ×.

To prepare the cryo-grid, 3 μl of the TIM/TIPIN protein complex was applied to a glow-discharged 400 mesh gold Quantifoil R1.2/1.3 holey carbon grid (Quantifoil Micro Tools, Q3100-CR1.3, Quantifoil R 2/1 300 mesh, Copper, Electron Microscopy Sciences), and subsequently vitrified using a fully automated Vitrobot Mark VI (FEI). Images were collected at liquid nitrogen temperature on a Titan Krios 300 kV field emission gun (FEG) electron microscope (Thermo Fisher Scientific/FEI), at a nominal magnification of 120,000× using a Falcon 3EC direct electron detector (Thermo Fisher Scientific) in counting mode, corresponding to a pixel size of 0.709 Å on the specimen level. In total, 2001 images with defocus values in the range of –0.5 μm were recorded with a dose rate of about 0.5 electrons per pixel per second. The total exposure time was set to 36 s per movie with intermediate fractions recorded every 0.6 s, resulting in an accumulated dose of about 43 electrons per Å^2^ over 60 fractions per movie stack. Data acquisition was conducted using the EPU automated data collection software (Thermo Fisher Scientific).

### Statistical analysis

The experiments were randomized and investigators were blinded to histological examination during all experiments. All statistical analyses were performed using Graphpad Prism 5.0 software (San Diego, CA, USA), with differences between groups considered significant with a *p* value *<* 0.001. Data are presented as mean values ± S. E. M. Histopathological scores and all other experimental data were compared using a *t*-test (two-sided) or one-way analysis of variance (ANOVA) followed by (post hoc) Newman-Keuls multiple and Turkey multiple comparison tests.

## SUPPLEMENTARY MATERIALS


