# Supplementary material for: Knock-down of the TIM/TIPIN complex promotes apoptosis in melanoma cells
Source: Oncotarget. 2020 May 19;11(20):1846–61. doi: 10.18632/oncotarget.27572 (PMC7244016; doi:10.18632/oncotarget.27572)
Supplement: Supplementary file 1 [file oncotarget-11-1846-s001.pdf]

Knock-down of the TIM/TIPIN complex promotes apoptosis in melanoma cells

SUPPLEMENTARY MATERIALS

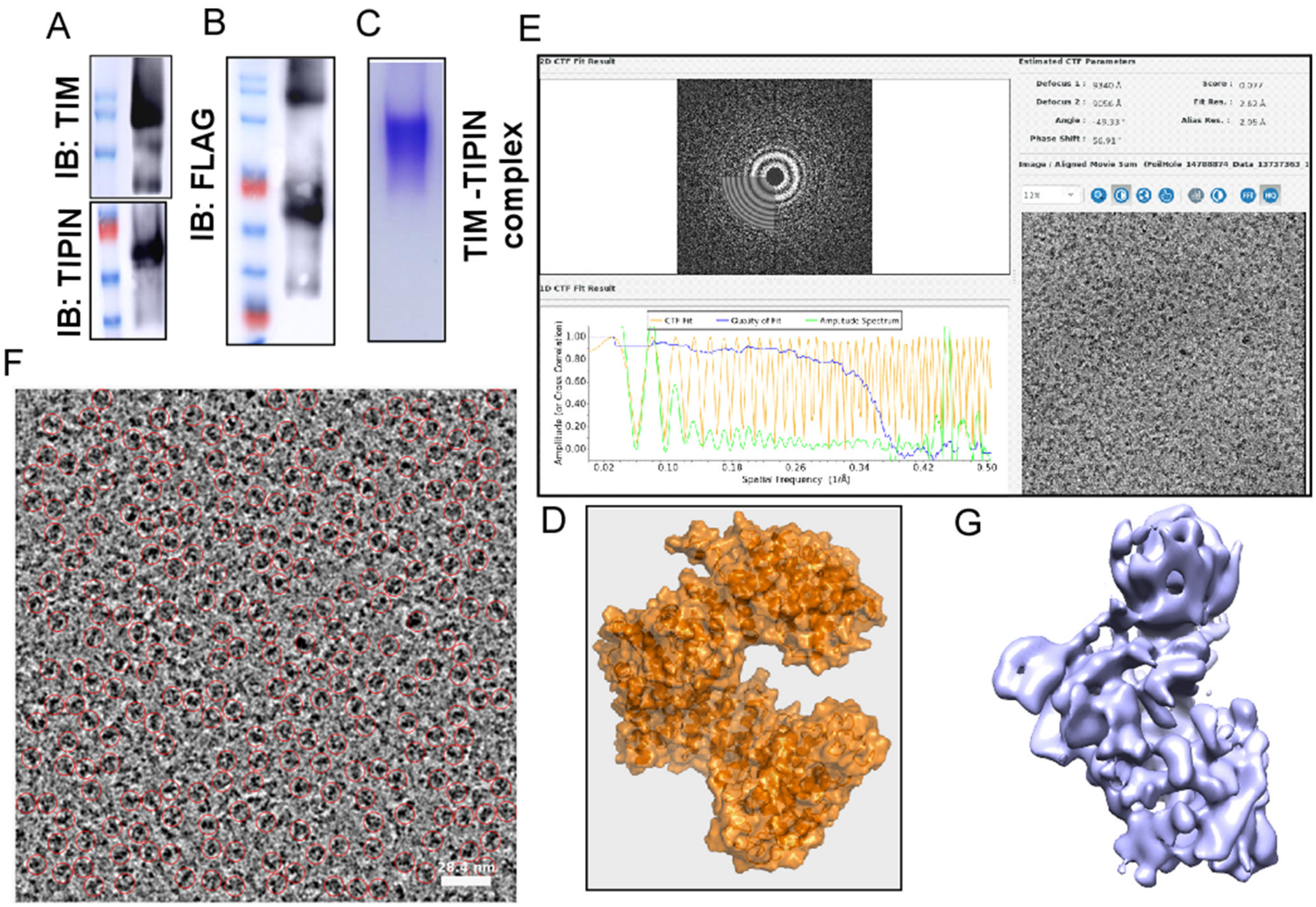

Supplementary Figure 1: (**A**, **B**) Immunoblotting of TIM-TIPIN purified protein complex with an anti-FLAG, anti-TIM, and anti-TIPIN antibody to confirm the protein complex. (**C**) Complex stability was confirmed by native page gel electrophoresis and Coomassie blue staining. (**D**) Prediction of a model of TIM and TIPIN structure using I-tasser online server (https://zhanglab.ccmb.med.umich.edu/I-TASSER/). A screenshot cryo-EM data processing (**E**) showing the thon ring, amplitude spectrum and quality of fit, and the micrograph itself. (**F**) particle picking image from cyro-EM data. A map (**G**) was generated using the Relion 3.0.6 software program.

Supplementary Table 1: Sequences shRNA

Gene	ID	Oligo ID	Catalog Num	Homologous	Accession	Sequence
TIM	Sh1	TRCN0000157211	RHS3979-98833334	Human	NM_003920	CCGGGCCCACACTAACCATTGCATTCTCGA GAATGCAATGGTTAGTGTGGGCTTTTTG
	Sh2	TRCN0000153090	RHS3979-98833358	Human	NM_003920	CCGGGCCGCATCATCAAGAACAATACTCGA GTATTGTTCTTGATGATGCGGCTTTTTG
TIPIN	Sh1	V2LHS_174170	RHS4430-99148704	Human	NM_017858	TGCTGTTGACAGTGAGCGACCTTAAGGCATG TATTTGATATAGTGAAGCCACAGATGTATAT CAAATACATGCCTTAAGGCTGCCTACTGCCTCGGA
	Sh2	V3LHS_382198	RHS4430-101034498	Human	NM_017858	TGCTGTTGACAGTGAGCGCCAGAAGAGCAAC AACAAAGAATAGTGAAGCCACAGATGTATTC TTTGTTGTTGCTCTTCTGTTGCCTACTGCCTCGGA

Supplementary Table 2: Sequences of qPCR primers

Target	Accession	Amplicon Length	Tm	F Primers 5′ -> 3′	R primer 5′ -> 3′
Human TIMELESS	NM_003920.5	120 bp	62°C	GAGACTTCTGCTCTGAGTTCC	CCAAGGCCCACATATAATAGGT
Human TIPIN	NM_017858.3	114 bp	62°C	CAGAGAGACAAGATGGTGAAGG	GAGCATCCAGCTTGGGTATATT
